# Association of Gut Microbiome Biomarkers With Mortality in Chinese Patients With Acute/Worsening Heart Failure

**DOI:** 10.1016/j.jacasi.2025.09.023

**Published:** 2025-12-02

**Authors:** Toru Suzuki, Hui Ding, Fang Yuan, Yiou Li, Jinqian Ren, Hong Zhan, Muhammad Zubair Israr, Xiangyang Sun, Zhaohui Qiu, Huiming Sheng

**Affiliations:** a Tongren Hospital Shanghai Jiao Tong University School of Medicine, Shanghai, China; b University of Leicester, Leicester NIHR Biomedical Research Centre and BHF Centre of Research Excellence, Leicester, United Kingdom; c Institute of Medical Science, The University of Tokyo, Tokyo, Japan; d Tellgen and Masswill Corporation, Shanghai, China; e School of Public Health, Bengbu Medical University, Bengbu, Anhui, China

**Keywords:** gut microbiome, heart failure, metabolite

The association of the gut microbiome with adverse outcomes of heart failure (HF) and use in risk stratification is a topic of recent interest. Investigations by some of the authors in various cohorts (eg, British,^1^ European,^2^^,^^3^ Japanese and South Asian populations^4^) have collectively shown that ethnic, geographical, and dietary differences affect the association of the gut microbiome to HF outcomes, and that a panel of markers of the choline/carnitine metabolism pathway, which includes trimethylamine-N-oxide (TMAO), shows greater association with adverse outcomes compared with TMAO alone.^5^, ^6^, ^7^ Previous investigations in the Chinese population had reported the association of TMAO as a single marker with adverse outcomes of chronic HF,^8^ acute myocardial infarction complicated by HF,^9^ and in aortic stenosis,^10^ but the current study is the first to assess the association of a panel of gut microbiome markers of choline/carnitine metabolism (choline, acetyl-L-carnitine, L-carnitine, TMAO, and γ -butyrobetaine) with adverse outcome in Chinese patients admitted with acute or worsening HF.

This single-center prospective observational study was undertaken at Tongren Hospital of Shanghai Jiao Tong University School of Medicine between August 2019 and January 2022 with institutional ethics approval (#2019-021-02). Consecutive consenting patients with age ≥18 years admitted for symptoms of new-onset or worsening HF were enrolled. Cardiac dysfunction as left ventricular ejection fraction of ≤40% and/or B-type natriuretic peptide (BNP) levels >400 pg/mL was also used as inclusion criteria. Exclusion criteria included previous history of cancer, renal dysfunction with estimated glomerular filtration rate (eGFR) ≤15 mL/min/1.73 m^2^, surgical procedure within the previous month, presence of sepsis, acute coronary syndrome in the previous 3 months, implantable cardiac defibrillator of cardiac resynchronization therapy within the previous 4 weeks, presence of acute myocarditis or hypertrophic obstructive or constrictive cardiomyopathy, and heart transplant recipient or admission for cardiac transplantation or left ventricular assist device implantation. Primary outcomes were mortality (all-cause) at 1 and 3 years as confirmed with government death records for all patients as a complete-case analysis. Patients were treated using standard contemporary Chinese guideline-based treatment.^11^ Blood sampling was done at admission as plasma collected in ethylenediaminetetraacetic acid (EDTA) tubes, separated by centrifugation, then frozen at −80°C until analysis was done as a single batch. Gut microbiome biomarkers were measured as previously described.^5^^,^^6^ The measured levels of the 5 gut microbiome biomarkers were dichotomized (elevated/not elevated) by the median value. Kaplan-Meier survival curve analysis and Cox proportional hazard analysis were used to analyze associations of gut microbiome biomarkers with mortality. Multicolinearity in the regression models was investigated by tolerance and variance inflation factor in linear regression. *P* values of <0.05 were considered significant.

A total of 229 patients were enrolled in the current study (mean 80 years, range 70-86 years; 55% [126 of 229] men) with follow-up of 4.24 years (median [IQR] 3.84-4.97 years). Demographic analysis showed higher prevalence for history of hypertension as etiology (72.1% [165 of 229], compared with history of ischemic heart disease at 45.4% [104 of 229]), and more lower-grade HF (NYHA functional class II 34.9% [80 of 229], III 58.1% [133 of 229], IV 7.0% [16 of 229]) compared with previously investigated populations^2^^,^^5^ (Supplemental Table 1). Kaplan-Meier survival curve analysis showed 15.3% mortality in the first year (35 of 229 patients, 95% CI: 11.2%-20.6%) and 32.3% at 3 years (74 of 229 patients, 95% CI: 26.7%-38.8%) (see Figure 1). All 5 of the investigated gut microbiome biomarkers were significantly elevated in patients who died at 1 and 3 years (*P* ≤ 0.013) (Supplemental Table 1). The most notable difference was >2-fold increase in TMAO levels in patients who died (7.7 [IQR: 4.7, 12.5] vs 3.4 [IQR: 2.1, 5.5] at 1 year, 7.2 [IQR: 4.4, 12.5] vs 3.4 [IQR: 2.2, 5.4] at 3 years, both *P* < 0.001, μmol/L) (Supplemental Table 1). The more markers being elevated showed stepwise increase with mortality at 1 and 3 years with a marked increase when all 5 markers were used (chi-square 8.38-25.47, *P* ≤ 0.004) (see Figure 1). BNP, the gold standard biomarker for diagnostic purposes and risk stratification of HF, showed association with death (HR: 1.55; 95% CI: 1.12-2.14 at 1 year; HR: 1.20; 95% CI: 0.97-1.49 at 3 years), which was slightly better with TMAO (HR: 1.78; 95% CI: 1.31-2.40 at 1 year; HR: 1.82; 95% CI: 1.45-2.30 at 3 years) but the 5 gut microbiome biomarkers when combined showed markedly increased association (HR: 3.37; 95% CI: 2.11-10.34 at 1 year; HR: 2.70; 95% CI: 1.79-7.80 at 3 years). Stepwise increase in associations was seen with number of elevated metabolites (chi-square, *P* < 0.001). HRs were adjusted for age, gender, BNP levels, and renal function by eGFR. Co-linearity was not seen in the multivariate model (tolerance 0.32-0.95, variance inflation factor 1.06-2.62).

**Figure 1:**
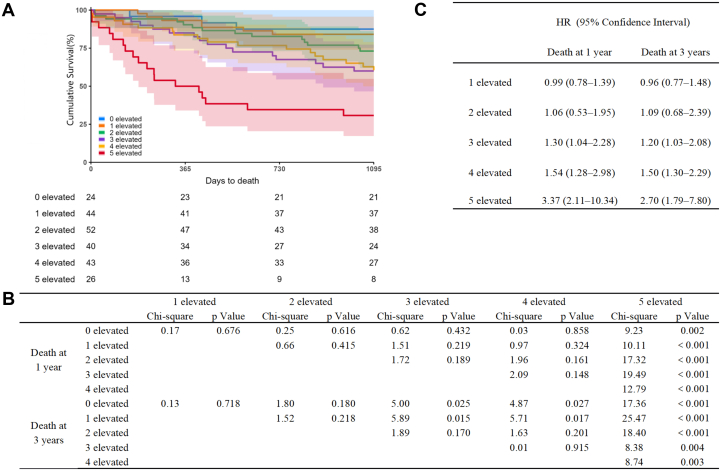
Association of Gut Microbiome Biomarkers With Mortality in Patients With Heart Failure (A) Kaplan-Meier survival curves stratified by the number of elevated gut microbiome biomarkers (0-5). Shaded areas indicate 95% CI; the number of events/deaths are shown below the x-axis. (B) Log-rank tests: chi-square values and *P* values comparing survival by the number of elevated biomarkers (pairwise comparisons vs 0 elevated and across groups) at 1 year and 3 years. (C) Adjusted HRs with 95% CIs from Cox proportional hazards models for mortality at 1 year and 3 years, comparing 1 to 5 elevated groups with 0 elevated (reference). Models are adjusted for age, sex, B-type natriuretic peptide, and renal function by estimated glomerular filtration rate.

The current study shows that gut microbiome biomarkers are associated with mortality in Chinese patients admitted with HF similar to previously investigated Caucasian populations despite different ethnic, geographical, and dietary backgrounds.^2^^,^^5^ One notable difference was that choline metabolism was more associated with adverse outcome, which differs from previous studies in Caucasian populations.^2^^,^^5^ This may reflect the increased dietary intake of choline in the Chinese population in the past decade^12^ with a trend for the Chinese diet switching from plant-based foods to animal-derived foods but also illustrates differences with Caucasians in which carnitine is more associated with HF outcomes.

In conclusion, the current study showed that the combined biomarkers of the choline/carnitine/TMAO-metabolic pathway comprise a potent panel of markers for risk stratification of HF, at least in admitted patients for stratification of 1- and 3-year mortality, in the Chinese population. This further supports the gut metabolite panel as a patient agnostic biomarker panel for HF outcomes. Limitations are that the current study was done at a single center with small sample size, and further investigations at multiple sites with larger sample size are needed to validate findings and assess the generalizability of the association of gut microbiome biomarkers for risk stratification of HF in the Chinese population, including geographical considerations. Risk stratification using biomarkers may serve as an adjunct tool for initiation and optimization of treatment of HF and warrants further consideration of clinical translation and adoption.^7^

## Funding Support and Author Disclosures

This work was supported by the following funding: Dr Suzuki: the Japan Heart Foundation, National Institute for Health Research (Leicester Biomedical Research Centre), the British Heart Foundation (BHF) including the Centre of Research Excellence Award (RE/24/130031), the Medical Research Council (MRC) UK Consortium on MetAbolic Phenotyping (MAP/UK), Grant-in-Aid for Scientific Research (A) (23H00454) and for Challenging Research (Pioneering) (22K18412) from the Japan Society for the Promotion of Science (JSPS), and the LeDucq Foundation. Dr Zhan: Shanghai Science and Technology Innovation Action Plan biomedical innovation and development project (No. 24S11902200). Dr Sheng: the National Natural Science Foundation of China (No. 82070730), the Fund of Shanghai Changning District Medical Doctor Innovation Talent Base (RCJD2021B01). The authors have reported that they have no relationships relevant to the contents of this paper to disclose.
