# Supplementary material for: Association of Gut Microbiome Biomarkers With Mortality in Chinese Patients With Acute/Worsening Heart Failure
Source: JACC Asia. 2025 Dec 2;5(12):1634–7. doi: 10.1016/j.jacasi.2025.09.023 (PMC12802354; doi:10.1016/j.jacasi.2025.09.023)
Supplement: Supplemental Table 1 [file mmc1.docx]

Supplemental Table 1. Patient demographics

|  | Overall  (n=229) | Death at 1 year (n=35) | Survival at 1 year (n=194) | p-Value | Death at 3 years (n=74) | Survival at 3 years (n=155) | p -Value |
| --- | --- | --- | --- | --- | --- | --- | --- |
| Age, years | 80 (70, 86) | 84 (74, 88) | 79 (69, 85) | **0.020** | 84 (74, 87) | 77 (69, 84) | **0.001** |
| Male, % (n) | 55 (126) | 40 (14) | 58 (112) | 0.053 | 50 (37) | 57 (89) | 0.292 |
| Systolic BP, mmHg | 132 (120, 152) | 130(110, 141) | 132 (120, 152) | 0.081 | 130 (117, 151) | 134 (120, 153) | 0.102 |
| Diastolic BP, mmHg | 78 (68, 85) | 77 (63, 80) | 78 (69, 87) | 0.102 | 74 (64, 81) | 78 (70, 89) | **0.012** |
| Heart rate, beats/min | 79 (67, 90) | 79 (61, 92) | 79 (68, 90) | 0.706 | 78 (64, 85) | 82 (70, 93) | **0.006** |
| Past history HF, % (n) | 42.4 (97) | 48.6 (17) | 41.2 (80) | 0.420 | 44.6 (33) | 41.3 (64) | 0.637 |
| Past history IHD, % (n) | 45.4 (104) | 31.4 (11) | 47.9 (93) | 0.072 | 45.9 (34) | 45.2 (70) | 0.911 |
| Past history diabetes, % (n) | 39.7 (91) | 42.9 (15) | 39.2 (76) | 0.683 | 37.8 (28) | 40.6 (63) | 0.685 |
| Past history hypertension, % (n) | 72.1 (165) | 62.9 (22) | 73.7 (143) | 0.189 | 70.3 (52) | 72.9 (113) | 0.679 |
| Smoker, % (n) | 13.1 (30) | 2.9 (1) | 14.9 (29) | 0.052 | 12.2 (9) | 13.5 (21) | 0.772 |
| Orthopnea, % (n) | 71.2 (163) | 88.6 (31) | 68.0 (132) | **0.014** | 78.4 (58) | 67.7 (105) | 0.097 |
| Oedema, % (n) | 50.2 (115) | 60.0 (21) | 48.5 (94) | 0.210 | 54.1 (40) | 48.4 (75) | 0.423 |
| Atrial fibrillation, % (n) | 48.5 (111) | 60.0 (21) | 46.4 (90) | 0.139 | 55.4 (41) | 45.2 (70) | 0.148 |
| Valvular heart disease, % (n) | 8.3 (19) | 5.7 (2) | 8.8 (17) | 0.548 | 8.1 (6) | 8.4 (13) | 0.943 |
| LVEF, % | 53 (40, 59) | 53 (41, 57) | 53 (38, 59) | 0.811 | 53 (40, 58) | 52 (38, 59) | 0.649 |
| HFpEF, LVEF≥50%, % (n) | 54.1 (124) | 54.3 (19) | 54.1 (105) | 0.811 | 55.4 (41) | 53.5 (83) | 0.649 |
| NYHA class II, % (n) | 34.9 (80) | 28.6 (10) | 36.1 (70) | **0.035** | 25.7 (19) | 39.4 (61) | **0.021** |
| III, %(n) | 58.1 (133) | 54.3 (19) | 58.8 (114) |  | 63.5 (47) | 55.5 (86) |  |
| IV, % (n) | 7.0 (16) | 17.1 (6) | 5.2 (10) |  | 10.8 (8) | 5.2 (8) |  |
| Creatinine, µmol/L | 96 (75, 122) | 121 (94, 139) | 90.0 (74, 117) | **0.003** | 117 (87, 144) | 84 (72, 111) | **0.000** |
| eGFR, mL/min/1.73m^2^ | 59 (42, 79) | 40 (33, 56) | 62 (45, 80) | **<0.001** | 43 (32, 60) | 66 (52, 84) | **0.000** |
| Uric acid, umol/L | 465 (351, 570) | 464 (376, 618) | 465 (346, 558) | 0.240 | 531 (369, 640) | 455 (347, 536) | **0.014** |
| Triglyceride, mmol/L | 1.0 (0.8, 1.3) | 1.0 (0.8, 1.2) | 1.1 (0.8, 1.4) | 0.399 | 1.0 (0.8, 1.3) | 1.1 (0.8, 1.4) | 0.528 |
| Cholesterol, mmol/L | 3.7 (3.1, 4.5) | 3.4 (2.6, 4.4) | 3.7 (3.1, 4.5) | 0.124 | 3.6 (3.2, 4.4) | 3.7 (3.1, 4.5) | 0.530 |
| HDL-C, mmol/L | 1.0 (0.8, 1.2) | 1.0(0.7, 1.2) | 1.0 (0.8, 1.2) | 0.667 | 1.0 (0.8, 1.3) | 1.0 (0.8, 1.2) | 0.618 |
| LDL-C, mmol/L | 2.4 (1.7, 3.1) | 1.9 (1.5, 2.8) | 2.4 (1.8, 3.2) | 0.059 | 2.3 (1.6, 2.8) | 2.4 (1.8, 3.2) | 0.231 |
| K^+^, mmol/L | 3.9 (3.5, 4.2) | 4.2 (3.7, 4.6) | 3.8 (3.5, 4.2) | **0.003** | 4.1 (3.6, 4.5) | 3.8 (3.5, 4.2) | **0.001** |
| Na^+^, mmol/L | 140 (138, 142) | 139 (136, 143) | 141 (138, 142) | 0.240 | 140 (137, 142) | 141 (138, 142) | 0.147 |
| Homocysteine, µmol/L | 18.7 (14.7, 26.6) | 21.0 (16.4, 28.6) | 18.4 (14.5, 24.8) | 0.085 | 21.1 (16.7, 29.9) | 17.9 (14.0, 23.1) | **0.001** |
| hs-CRP, mg/L | 6.3 (2.4, 20.5) | 6.9 (3.9, 24.1) | 6.2 (2.1, 20.3) | 0.174 | 6.8 (3.1, 16.0) | 6.2 (2.2, 20.5) | 0.484 |
| HbA1c, % | 6.1 (5.7, 6.9) | 6.3 (5.6, 7.0) | 6.1 (5.7, 6.8) | 0.611 | 6.1 (5.7, 7.0) | 6.2 (5.8, 6.8) | 0.485 |
| Hemoglobin, g/L | 123 (107, 141) | 112 (97, 129) | 125 (109, 143) | **0.006** | 115 (101, 129) | 127 (111, 146) | **0.001** |
| B-type natriuretic peptide, pg/mL | 969 (591, 1867) | 1297 (811, 3601) | 908 (555, 1677) | **0.004** | 1142 (660, 2011) | 856 (558, 1795) | **0.030** |
| Trimethylamine N-oxide, µmol/L | 4.2 (2.5, 7.4) | 7.7 (4.7, 12.5) | 3.4 (2.1, 5.5) | **<0.001** | 7.2 (4.4, 12.5) | 3.4 (2.2, 5.4) | **0.000** |
| Acetyl-L-carnitine, µmol/L | 12.4 (9.1, 17.6) | 16.9 (11.3, 24.2) | 11.9 (8.9, 16.6) | **0.004** | 15.6 (11.0, 23.3) | 11.4 (8.8, 15.1) | **0.001** |
| L-carnitine, µmol/L | 47.1 (37.6, 60.2) | 58.7 (44.6, 80.6) | 45.3 (36.8, 59.1) | **0.008** | 54.4 (39.6, 67.2) | 44.6 (35.3, 58.0) | **0.010** |
| γ-butyrobetaine, µmol/L | 1.2 (0.9, 1.8) | 1.5 (1.1, 2.1) | 1.2 (0.9, 1.7) | **0.009** | 1.5 (1.0, 2.0) | 1.2 (0.9, 1.6) | **0.001** |
| Choline, µmol/L | 17.0 (13.5, 21.4) | 18.9 (16.0, 26.0) | 16.3 (13.1, 20.9) | **0.008** | 17.9 (15.1, 23.5) | 16.1 (12.7, 20.8) | **0.013** |

Data are reported as median (interquartile range) for continuous variables and as a percentage for categoricalvalues. LVEF, Left Ventricular Ejection Fraction; HFpEF, Heart Failure with Preserved Ejection Fraction; NYHA, New York Heart Association; eGFR, Estimated Glomerular Filtration Rate; HDL-C, High Density Lipoprotein Cholesterol; LDL-C, Low Density Lipoprotein Cholesterol; hs-CRP, High Sensitivity C-Reactive Protein.
